# Investigating secondary white matter degeneration following ischemic stroke by modelling affected fiber tracts

**DOI:** 10.1016/j.nicl.2022.102945

**Published:** 2022-01-22

**Authors:** Ivana Kancheva, Floor Buma, Gert Kwakkel, Angelina Kancheva, Nick Ramsey, Mathijs Raemaekers

**Affiliations:** a UMC Utrecht Brain Center, Department of Neurology and Neurosurgery, University Medical Center Utrecht, PO Box 85060, 3508AB Utrecht, The Netherlands; b Department of Anatomy and Neurosciences, MOVE Research Institute Amsterdam, VU University Medical Center, PO Box 7057, 1007MB Amsterdam, The Netherlands; c Department of Rehabilitation Medicine, VU University Medical Center, PO Box 7057, 1007MB Amsterdam, The Netherlands

**Keywords:** Ischemic stroke, Secondary degeneration, Diffusion tensor imaging, DTI simulation, Tractography database, Motor deficit

## Abstract

Secondary white matter degeneration is a common occurrence after ischemic stroke, as identified by Diffusion Tensor Imaging (DTI). However, despite recent advances, the time course of the process is not completely understood. The primary aim of this study was to assess secondary degeneration using an approach whereby we create a patient-specific model of damaged fibers based on the volumetric characteristics of lesions. We also examined the effects of secondary degeneration along the modelled streamlines at different distances from the primary infarction using DTI. Eleven patients who presented with upper limb motor deficits at the time of a first-ever ischemic stroke were included. They underwent scanning at weeks 6 and 29 post-stroke. The fractional anisotropy (FA), mean diffusivity (MD), primary eigenvalue (*λ*_1_), and transverse eigenvalue (*λ*_23_) were measured. Using regions of interest based on the simulation output, the differences between the modelled fibers and matched contralateral areas were analyzed. The longitudinal change between the two time points and across five distances from the primary lesion was also assessed using the ratios of diffusion quantities (rFA, rMD, r*λ*_1_, and r*λ*_23_) between the ipsilesional and contralesional hemisphere. At week 6 post-stroke, significantly decreased *λ*_1_ was found along the ipsilesional corticospinal tract (CST) with a trend towards lower FA, reduced MD and *λ*_23_. At week 29 post-stroke, significantly decreased FA was shown relative to the non-lesioned side, with a trend towards lower *λ*_1_, unchanged MD, and higher *λ*_23_. Along the ipsilesional tract, the rFA diminished, whereas the rMD, r*λ*_1_, and r*λ*_23_ significantly increased over time. No significant variations in the time progressive effect with distance were demonstrated. The findings support previously described mechanisms of secondary degeneration and suggest that it spreads along the entire length of a damaged tract. Future investigations using higher-order tractography techniques can further explain the intravoxel alterations caused by ischemic injury.

## Highlights

• Secondary white matter degeneration was studied in 11 ischemic stroke patients.
• We used a custom-developed approach to model damaged fibers associated with a lesion.
• This approach tackles the inter-subject variability in lesion size and location.
• Findings suggest that secondary degeneration spreads along an entire fiber’s length.

## 1 Introduction

Ischemic stroke disrupts cerebral white matter, which can cause disability (Langhorne et al., 2011). Following injury, secondary degeneration occurs, defined as neurodegeneration of the proximal and distal parts of axons (Egorova et al., 2020). Secondary degeneration is divided into anterograde (or Wallerian) and retrograde. Anterograde degeneration proceeds from the primary stroke area in the direction of the axon’s terminals (Pierpaoli et al., 2001), whereas retrograde degeneration advances proximally towards the cell body (Jindahra et al., 2012). Within days after injury, axonal structures start to disintegrate, followed by myelin degradation, cytokine upregulation and macrophage infiltration after several weeks, and finally, fibrosis and atrophy of the affected tracts (Johnson et al., 1950, Lampert and Cressman, 1966).

Secondary degeneration of the pyramidal tract is a well-known phenomenon after motor pathway infarction, which can be characterized using diffusion tensor imaging (DTI) (Thomalla et al., 2004, Thomalla et al., 2005, Werring et al., 2000). DTI can quantify white matter integrity using the parameters of fractional anisotropy (FA; scalar measure of directional bias ranging from 0 to 1), mean diffusivity (MD; average molecular diffusion), axial diffusivity (AD; diffusion parallel to fibers, equivalent to the primary eigenvalue *λ*_1_), and radial diffusivity (RD; diffusion perpendicular to fibers, an average of the medium and minimum eigenvalues *λ*_23_) (Basser and Pajevic, 2003, Pierpaoli and Basser, 1996). In an undamaged white matter tract, water diffuses rapidly along the principal directions of axons and more slowly perpendicular to them, resulting in anisotropic diffusion. In contrast, pathological processes alter diffusion, making DTI a suitable modality to study structural degeneration of long descending tracts after stroke (Werring et al., 2000).

Several studies have examined secondary degeneration of the pyramidal tract using DTI (Liang et al., 2007, Pierpaoli et al., 2001, Radlinska et al., 2010). For example, Thomalla et al. (2004) assessed anterograde degeneration in 9 ischemic stroke patients and found decreased FA and *λ*_1_, unchanged MD, and increased *λ*_23_ within 2 weeks after stroke onset. Significant FA decreases have also been demonstrated from week 1 to week 12 post-stroke upstream and downstream from the primary lesion in patients with pontine infarction (Liang et al., 2008). A similar dynamic pattern was revealed by Yu and colleagues (2009) who reported that diffusion quantities fluctuated during the first 3 months following injury and then stabilized. Concerning retrograde degeneration, time-dependent optic tract degeneration has been identified after occipital lobe damage (Cowey et al., 2011, Jindahra et al., 2012). One of the first reports involving the corticospinal tract (CST) discovered upstream degeneration after a pontine lesion, which extended to the cerebral peduncle and internal capsule four years following infarction (Kobayashi et al., 2005). These findings indicate the presence of continuous secondary degeneration remote from a lesion. However, data on further distal changes are limited (Wang et al., 2016) and the spatio-temporal progression of the process remains elusive.

In the present investigation, we introduce a custom-developed approach to quantify axonal injury caused by secondary degeneration. Using a DTI tractography database of healthy volunteers, we can model damaged fibers based on the volumetric lesion characteristics of individual patients. In principle, this method has several advantages. First, it considers the inter-subject variability in lesion size and location, and thereby, effectively handles the heterogeneity pertinent to stroke populations. Second, by using a tractography database of healthy participants to examine secondary degeneration, our approach circumvents several challenges associated with current procedures for region of interest (ROI) definition. One of them is the imprecision and laboriousness of manual tract delineation in pure ROI analysis (Froeling et al., 2016), which has been adopted in several former examinations of secondary degeneration (Liang et al., 2007, Liang et al., 2008, Thomalla et al., 2004, Thomalla et al., 2005, Yu et al., 2009). While being easy to perform, this approach requires good anatomical knowledge to carefully determine the ROIs position given the often-ill-defined boundaries of fiber tracts (Froeling et al., 2016). Other shortcomings of commonly employed ROI methods include the ambiguity in the selection of pathways and tracking parameters, such as seeding and stopping criteria in tract ROI approaches (Knösche et al., 2015, Maier-Hein et al., 2017, Schilling et al., 2019), the sequence of ROI positioning, which can compromise tract reconstruction accuracy (Hattingen et al., 2009), or the tract displacement and edema in the vicinity of lesions (Jellison et al., 2004). Recently, some solutions have been designed to estimate how focal lesions might exert pathological effects distally from the primary site of infarction (e.g., Foulon et al., 2018, Griffis et al., 2021). These tools have enabled the study of stroke as a network connectivity disorder and have been applied for various purposes: to identify white matter damage in the context of stroke aphasia (Forkel and Catani, 2018), to relate structural disconnection to post-stroke behavioral deficits (Salvalaggio et al., 2020) or executive dysfunction (Hobden et al., 2021), or to reveal links between white matter connections and the brain’s functional segregation (de Schotten et al., 2020).

In this study, we use our approach for the identification of more restricted damage to specific fiber tracts, offering an alternative method to investigate secondary degeneration along entire modelled pathways, as well as at several distances from the primary lesion. In addition, we assess the output of modelled streamlines in relation to the neurological deficit observed in our study population. Using a non-invasive procedure to map fibers where degeneration is expected to occur has important clinical implications. It can illustrate the potential of studying white matter degeneration as a prognostic marker of stroke-induced symptoms, which may be more reliable than lesion size or location alone. Estimating damaged streamlines can also contribute knowledge about the extent of structural pathology, providing a comprehensive set of affected voxels for finding correlations with functional outcomes. In turn, this has the potential to aid prognosis about long-term post-stroke impairment (Lin et al., 2019) and treatment response (Puig et al., 2017).

First, we created a patient-specific model of the fibers affected by a stroke to maximize our sensitivity and then we examined the effects of secondary degeneration on the DTI signals in the simulated white matter regions.

## 2 Methods

### 2.1 Subjects

Fourteen ischemic stroke patients were recruited as part of the EXPLICIT-stroke trial from 2009 to 2012. This was a Dutch translational research program including two multi-center single-blinded randomized trials aimed at elucidating the mechanisms that underlie post-stroke recovery of upper extremity (details of the design and interventions are described in Kwakkel et al., 2008). All patients presented with mild-to-moderate upper limb paresis following a clinically primary brain infarction and had high probability of return of dexterity. They were enrolled in the ‘favorable prognosis’ group within one of the trial’s projects, which involved motor functional magnetic resonance imaging (fMRI) tasks and detailed kinematic measurements of upper limb movements, where a full paresis would have resulted in non-compliance with the research protocol. The timepoints were chosen within the context of the trial and represent an interval during which clinical changes are observed, and effects of secondary degeneration are anticipated. Patients were included if they (a) had experienced a first-ever ischemic stroke that resulted in hospitalization, verified by computer tomography (CT) or MRI examination; (b) presented with upper extremity mono- or hemiparesis at stroke onset, determined by a National Institute of Health Stroke Score (NIHSS) of 4 points or less on item 5 (Brott et al., 1989); (c) were aged between 18 and 80 years; (d) were able to understand instructions as indicated by a Mini-Mental State Examination (MMSE) score of 23 or higher (Folstein et al., 1975); (e) were able to provide written consent for participation. Exclusion criteria were: (a) inability to perform flexion–extension finger movements or reach-to-grasp movements with the paretic upper limb in week 6 post-stroke; (b) orthopedic restrictions of upper extremities; (c) pacemakers or other metallic implants incompatible with the MRI scanner; (d) botulinum toxin injections or other medication that may affect upper limb function; (e) communication restrictions as denoted by a score of 3 or less on the Utrecht Communication Observation (UCO) (Pijfers et al., 1985). Three subjects were excluded due to uncertainties about pre-existing pathology, namely: one has suffered a hemorrhage close to the area of infarction; another patient had a prominent subcortical, as well as cortical lesion, and we could not determine which one resulted in hospitalization; a third patient exhibited pathology consistent with cortical laminar necrosis. All included patients were Dutch nationals, without former neurological or neuropsychiatric conditions. Consent forms were obtained in accordance with the Declaration of Helsinki (2013). Patients’ characteristics are summarized in Table 1. To create the tractography database for modelling damaged streamlines in patients, we also used data of a control group of 78 subjects of comparable age. Healthy participants were required to not have a history of neurological and/or psychiatric illnesses, to be aged between 18 and 80 years, and to not have any metallic implants incompatible with the scanner’s environment. Data of the control group were collected as part of the creation of an fMRI and DTI database for BrainCarta B.V. (Utrecht, The Netherlands; https://braincarta.com/).

**Table 1:** Patient characteristics at weeks 6 and 29 after stroke (n=11).

**Patient**	**Age (years)**	**Sex**	**Ipsilesional hemisphere**	**Lesion location**	**Lesion volume (mm^3^)**	**FMA-UE week 6**	**FMA-UE week 29**
1	64	M	R	IC, BG, CR	11.510	62	63
2	71	M	L	P	426	65	66
3	73	M	L	BG	5432	46	61
4	63	M	R	P	218	51	65
5	66	M	R	BG	7877	63	61
6	59	M	L	Thalamus, IC	360	62	65
7	60	M	R	IC, extending to cortex	3296	57	60
8	37	F	R	BG	1669	59	63
9	54	M	R	IC, BG, CR	1037	44	57
10	57	F	R	BG	5961	54	56
11	45	M	R	P	920	14	58
Mean	59						
SD	10.68						
**Total**		**2F, 9M**	**3L, 8R**	**3P, 8SC**			

*Note.* F, female; M, male; L, left; R, right; BG, basal ganglia; CR, corona radiata; IC, internal capsule; P, pontine; SC, subcortical. FMA-UE, upper extremity section of the Fugl-Meyer Assessment of Motor Recovery after Stroke.

### 2.2 Scanning protocol

Images were acquired on two Philips Achieva 3.0 Tesla MRI scanners (Philips Healthcare, Eindhoven, The Netherlands) located at the University Medical Center Utrecht (UMCU) and at the Leiden University Medical Center (LUMC). Seven patients were recruited from hospitals near Utrecht and scanned at the UMCU, and the other four were recruited near Leiden and scanned at the LUMC. The scanner model, imaging protocol, and quality control parameters across the two sites were identical. Patients’ T1-weighted images and diffusion MRI images were collected at week 6 (time point 1) and week 29 (time point 2) post-stroke. Control subjects all underwent scanning at the UMCU. The head was fixed by vacuum fixation cushions before scanning. High-resolution whole-brain anatomical scans were obtained for all participants for anatomical reference (3D T1-weighted scan: TR = 9806 ms; TE = 4.59 ms, flip angle = 8°, 140 slices, 0.875 × 0.857 × 1.2 mm, FOV(AP, FH, RL) = 224 × 168 × 177 mm).

Diffusion-weighted imaging (DWI) data were acquired in transverse orientation using parallel imaging sensitivity encoding (SENSE) (p reduction = 2). For patients, the following parameters were used: TR = 8481 ms; TE = 60 ms, FOV(AP, FH, RL) = 224 × 120 × 224 mm; voxel size = 2.00 × 2.00 × 2.00; 60 slices; 32 diffusion gradients; b = 800 s/mm^2^. Data of the control group were collected outside the trial with a different imaging protocol. While two complete DWI datasets were obtained for the control subjects, as opposed to a single set for the patients at each measurement point, the other acquisition parameters did not substantially differ. Parameters of the sequence for obtaining diffusion-weighted images in control subjects were: TR = 7110 ms; TE = 69 ms, FOV(AP, FH, RL) = 240 × 150 × 240 mm; isotropic voxel size = 1.88 × 1.88 × 2.00 mm; 75 slices; 32 diffusion gradients; b = 1000 s/mm^2^; 2 series with opposing phase-encoding blip (ap/pa). Slices were acquired with no inter-slice gap. All diffusion series included a reference image without diffusion weighting as well (b = 0 s/mm^2^).

### 2.3 Simulation approach

To define the specific ROIs where secondary degeneration effects would be expected in the diffusion data of the patients, we superimposed lesion volumes on the tractograms of control subjects and selected streamlines passing through each lesion. This required the creation of (1) lesion segmentation volumes of the patients and (2) tractograms of the control subjects in Montreal Neurological Institute (MNI) space.

#### 2.3.1 Lesion segmentation on Patients’ images

**Fig. 1:**
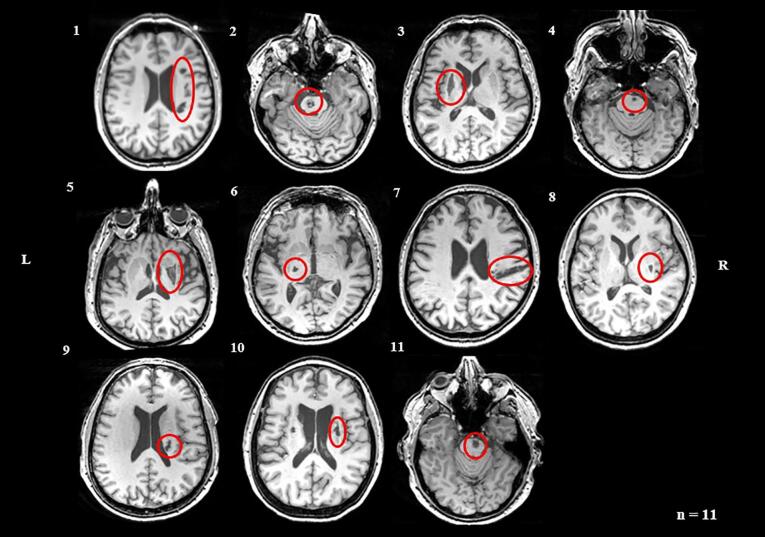
Axial structural T1-weighted anatomical scans at the level of maximum infarct volume for each stroke patient obtained at week 29 post-stroke. Arabic numbers denote the case numbers of patients. L = left; R = right.

Lesions were manually delineated on each slice of subjects’ T1-weighted images using ITK-Snap 3.6.0. (www.itksnap.org) by two researchers blinded to patients’ former medical history (primary rater I.K.; secondary rater A.K.), according to the protocol described by Yushkevich et al. (2006). The anatomical scans from the second time point were used to capture any tissue degeneration occurring between the two measurements. Any uncertainties regarding lesion location and extent were discussed with a third expert (M.R.). Segmentation reproducibility was determined using the Dice Similarity Coefficient (DSC), where a score of 0 indicates no spatial overlap and a score of 1 denotes perfect match (Crum et al., 2006). High correspondence was shown between the segmentations of the two raters (DSC = 0.76). Fig. 1 displays the lesion locations in all included patients.

#### 2.3.2 Lesion volumes post-processing

Next, the native space T1-weighted patient images from time point 2 and the corresponding lesion maps were coregistered to the b = 0 reference images from time point 1. Subsequently, the coregistered lesion volumes were spatially normalized to the elderly template in MNI space of the Clinical toolbox integrated in SPM12 (Statistical Parametric Mapping, https://www.nitrc.org/projects/clinicaltbx/), which is specifically designed for normalizing structural scans from older people, including those with brain injury (Rorden et al., 2012). Regularization was reduced by an order of magnitude to ensure proper fit of the ventricles and surrounding white matter. Quality checks of the registration and normalization procedures were performed by visual inspection. Then, the generated streamline and distance volumes were inverse normalized back to native space, and resliced to the volume and resolution of the diffusion scans. The resulting images were used for all subsequent statistical analyses.

#### 2.3.3 Creation of tractograms of control subjects

Initially, the DWI images were denoised (Veraart et al., 2016a, Veraart et al., 2016b) and geometry corrected using the b = 0 images of the series with opposed traversal of k-space in the phase-encoding direction (Andersson et al., 2003). Both series included a single b0 image. Then, DWI data were corrected for eddy currents and head motion (Andersson and Sotiropoulos, 2016) using the multi-processor variant of FSL's ‘eddy’. A mean b0 image was calculated and stored as a reference for registering other images to the DWIs. A response function for constrained spherical deconvolution (CSD) was created based on the diffusion data on every individual subject (Dhollander et al., 2016, September; Dhollander et al., 2018, June) and subsequently applied for estimating the fiber orientation distribution (FOD) of each voxel (Tournier et al., 2004). We created our response function per subject to optimize the validity of each individual tractography.

Five million streamlines were generated for each subject using a deterministic CSD algorithm (Tournier et al., 2012) with the following parameters: step size of 0.1 times the voxel size; minimum streamline length of 5 times the voxel size; tracking cut-off at an FOD of 0.1; maximum angle of 9 degrees per step. Seeds were selected randomly throughout the brain. To improve the biological plausibility of the tractograms, the 5 million streamlines were filtered down to 1 million, so that the FOD lobe integrals would better match the streamline densities (Smith et al., 2013). To normalize the tractograms to MNI space, they were first registered to the structural images applying the parameters derived from the registration procedure using the b = 0 images. Displacement maps for moving the tractogram coordinates to MNI space were obtained based on the T1-weighted images and spatially normalized with the ‘unified segmentation’ procedure in SPM12 (Ashburner and Friston, 2005), using the same regularization settings and template as for the patients.

#### 2.3.4 Modelling damaged fibers in patients

The fibers predicted to be damaged by a specific stroke were selected by including streamlines from the tractograms for which at least one coordinate was positioned within a voxel marked as part of a lesion. These streamlines were mapped onto a volume representing the number of affected fibers passing through each voxel. For each lesion, this process was repeated for every tractogram of the control subjects, resulting in 75 volumes per patient. An average was produced of these 75 volumes, representing the final model of damaged fibers in a given patient. Note that two simulation models were created for every patient, each based on the normalized lesion segmentation volumes of one of the two raters. The DSC was used to evaluate the similarity between the generated output images. Inter-rater agreement was excellent (DSC = 0.89), suggesting high reproducibility of the models. Thus, we continued only with the images of the primary rater (I.K.).

**Fig. 2:**
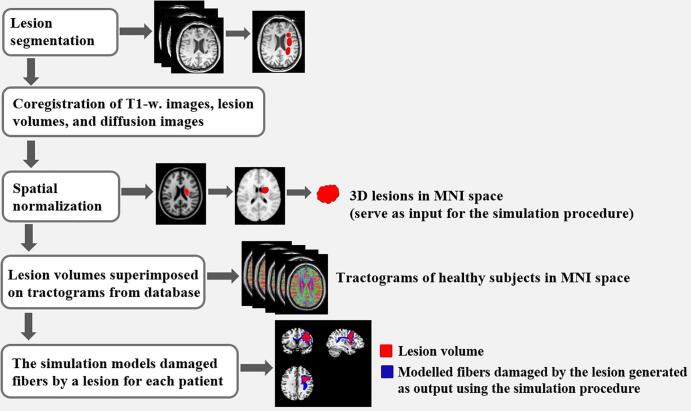
Schematic representation of the simulation pipeline. Lesion areas are marked in a 3D volume for every patient and then projected onto MNI space. The normalized lesion volumes serve as input for the simulation. During the procedure, all the streamlines that pass through an infarct area are selected to generate a prediction model comprising voxels with damaged fibers.

Furthermore, an additional volumetric output was created for every patient, which included the corresponding distance from a lesion along the length of the streamlines for every voxel containing at least a single affected streamline. The latter output was also the average of all simulations (n = 75) in the control subjects and was used to assess a possible degenerative process advancing upstream or downstream from the respective lesion location. Fig. 2 depicts a schematic representation of the simulation pipeline.

### 2.4 Diffusion data pre-processing

Diffusion images of patients were denoised and corrected for motion artifacts and eddy currents using the same algorithms as for the control subjects. After that, tensor models were fitted for each subject’s first and second time point scan and maps of FA, MD, *λ*_1_, and *λ*_23_ were generated with the MRtrix3 package (Tournier et al., 2019). The diffusion tensor of each voxel was calculated, and the eigenvalues were extracted. From them, the FA, MD, and RD (*λ*_23_) were computed according to the following equations:

$$\mathrm{FA}=\sqrt{\frac{{\left(\lambda_{1}-\lambda_{2}\right)}^{2}+{\left(\lambda_{1}-\lambda_{3}\right)}^{2}{\left(\lambda_{2}-\lambda_{3}\right)}^{2}}{2{\left(\lambda_{1}+\lambda_{2}+\lambda_{3}\right)}^{2}}}$$

$$\mathrm{MD}=\frac{\lambda_{1}+\lambda_{2}+\lambda_{3}}{3}$$

$$\mathrm{RD}=\frac{\lambda_{2}+\lambda_{3}}{2}$$

In addition, for each patient, the b = 0 image from time point 2 was coregistered and resliced to the b = 0 reference image from time point 1 using a rigid body transformation. The derived transformation matrix was applied to the corresponding volumes including the diffusion parameters using SPM12 (https://www.fil.ion.ucl.ac.uk/spm/software/spm12/). The resulting images were normalized to MNI space using the voxel displacement maps that were previously established with the Clinical toolbox.

### 2.5 Group-wise analysis for modelling damaged fibers

To assess if the modelling procedure would produce results consistent with the upper limb deficit exhibited in the study population, we performed a group-wise analysis using the volumetric images from the simulation’s output, comprising the number of damaged streamlines passing through each lesion voxel, as input. For this analysis, the Statistical nonParametric Mapping (SnPM) toolbox was employed (SnPM13.1.06, http://www.nisox.org/Software/SnPM13/) (Winkler et al., 2014). SnPM provides a framework for voxel-wise inference using a non-parametric multiple comparisons method with permutations. It uses the General Linear Model to construct pseudo *t*-statistic images, which are then assessed for significance using a standard non-parametric multiple comparisons procedure based on permutation testing. This approach was chosen due to the non-Gaussian distribution of the simulation output at the voxel level when comparing across patients.

**Fig. 3:**
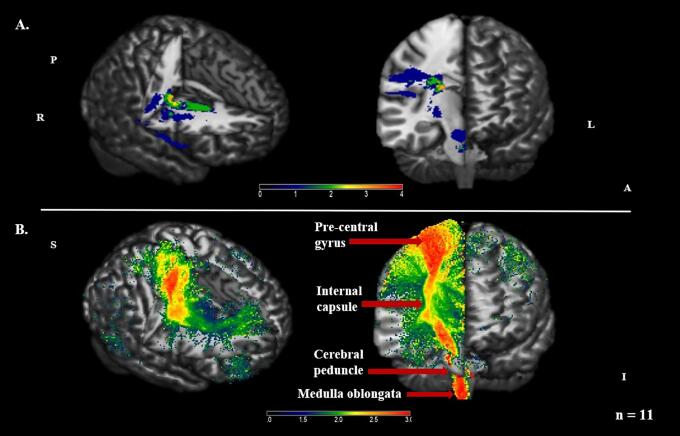
Lesion prevalence and modelled damaged fibers in patients (n = 11) visualized using MRIcron V2016. *Panel A.* Distribution of voxels affected by a lesion. The color bar indicates the number of subjects with a lesion in each voxel. *Panel B.* SnPM pseudo-T map displaying the results for the group-mean model comprising damaged fibers. The simulated streamlines map onto the CST, which carries movement-related information from the cerebral cortex to the brainstem. The color bar illustrates the number of subjects for whom fibers will be damaged by a lesion in each voxel. A = anterior; I = inferior; L = left; P = posterior; R = right; S = superior. Group average based on a threshold of *p* < .05 (FWE).

First, the damaged streamline models for patients with a right-sided motor deficit were flipped along the x-axis. This ensured that the assumed lesioned hemisphere corresponded to the right side of the brain for all subjects. Then the images were entered into SnPM, and a one sample *t* test was carried out with a threshold of *p* < .05 (family-wise error, FWE) based on 2048 permutations. According to the results, the modelled fibers mapped onto the CST to a large extent, as would be expected considering the hand mono- or hemiparesis that patients presented with at stroke onset (Fig. 3).

### 2.6 Tract region of interest-based analysis

**Fig. 4:**
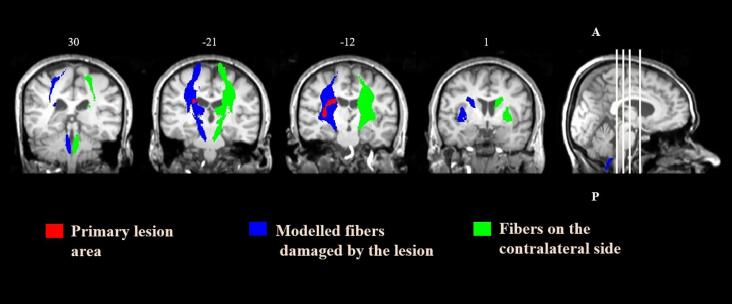
Modelled damaged fibers for a patient with right striatocapsular infarction, coronal slices. Red areas show the primary lesion. Blue areas illustrate the ROI in the ipsilesional hemisphere comprising the modelled damaged fibers. Green areas visualize the reference ROI on the contralateral side. ROIs are overlaid onto the patient’s coregistered T1-weighted image from week 29 post-stroke. Numbers above the slices denote Z coordinates. A = anterior; P = posterior. (For interpretation of the references to color in this figure legend, the reader is referred to the web version of this article.)

Next, a tract ROI-based analysis was conducted on the diffusion parameters in the ipsilesional (side of the lesion) and contralesional (opposite side of the lesion) hemisphere. To establish the ROI where degeneration was expected to occur, we thresholded the modelled output streamlines (>3 damaged fibers per voxel). Voxels inside the primary lesion or where the FA was < 0.1 were excluded. The contralateral ROI was used as a reference area, which was generated by flipping the left–right orientation of the simulation output (Fig. 4). Values of FA, MD, *λ*_1_, and *λ*_23_ were obtained by averaging across all voxels within each ROI. In addition, for each diffusion metric and time point, the ratio (rFA, rMD, r*λ*_1_, and r*λ*_23_) between the ipsilesional and contralesional side was calculated (e.g., rFA = FA_ipsilesional side_/FA_contralesional side_). This is a common approach in DTI studies investigating secondary degeneration, based on the premise that diffusion indices along tracts on the left and right sides do not differ, and that their values in the non-lesioned hemisphere remain stable after stroke (e.g., Pierpaoli et al., 2001, Thomalla et al., 2005, Yu et al., 2009). To confirm whether this condition was met, we assessed the fluctuation in diffusion quantities on the contralesional side prior to using the ratios. Then the rFA, rMD, r*λ*_1_, and r*λ*_23_ of the ROI of the ipsilesional tract between the two time points were compared to examine any longitudinal change. Diffusion parameters and their respective ratios were also computed for the ROI of the primary infarction.

### 2.7 Distance-based region of interest analysis

Furthermore, to investigate the spatio-temporal changes along the ipsilesional tract, the original ROI was divided into five regions representing segments of the damaged fibers at several distances from the primary infarction area. The included distances were 0–10, 10–20, 20–30, 30–40, and 40–50 mm from the primary lesion, as calculated along the length of the streamlines. The ratios (rFA, rMD, r*λ*_1_, and r*λ*_23_) between the ipsilesional and contralesional hemisphere were computed for each distance and compared between the two time points.

### 2.8 Statistical analysis

Statistical analysis was performed using SPSS 25.0. for Windows (SPSS Inc., Chicago, IL). Side differences, as well as differences between the two time points, were analyzed using paired samples *t* tests for normally distributed data, and Wilcoxon signed-rank tests when the normality assumption was violated. To study the changes in diffusion quantities along the degenerating tract at different distances from the primary lesion, a two-way repeated measures ANOVA was carried out on the rFA, rMD, r*λ*_1_, and r*λ*_23_ of the ipsilesional ROIs. Distance (6 levels) and time point (2 levels) were inserted as within-subject factors. The significance level was defined as 2-tailed and the threshold was set at *p* = .05 for all statistical procedures.

## 3 Results

### 3.1 Differences between diffusion indices along the ipsilesional and contralesional tract

To assess any diffusion abnormalities along the damaged streamlines at week 6 post-stroke, we examined the differences between the diffusion indices in the ROIs of the lesioned and non-lesioned hemisphere. Then we carried out the same analysis on the parameters from week 29. Table 2 shows the FA, MD, *λ*_1_, and *λ*_23_ on both sides at each time point. At week 6 post-stroke, the *λ*_1_ of the ROI of the ipsilesional tract was significantly lower than the *λ*_1_ of the contralesional ROI. At week 29, the FA of the ROI of the ipsilesional tract was significantly lower than the FA of the contralesional tract. The differences between the two sides for the remaining comparisons of interest did not reach statistical significance.

**Table 2:** Comparisons of diffusion parameters between the ipsilesional and contralesional side at two time points post-stroke in patients (n=11).

**Tract ROI Analysis**					
					**Group comparison, *P* value**
**Diffusion parameter**	**Time post-stroke**	**Ipsilesional side (IS)**	**Contralesional side (CS)**	**Test statistics**	**IS vs CS**
**FA (dimensionless)**	W6 **W29**	0.40±0.07 **0.39±0.06**	0.43±0.06 **0.43±0.07**	*t*(10) = 1.546 ***t*(10) = 2.382**	0.153 **0.038**
**MD (×10^−3^mm^2^/sec)**	W6 W29	0.92±0.12 0.97±0.12	0.95±0.15 0.96±0.18	T = 24; z = -0.80† *t*(10) = -0.434	0.424**^†^** 0.674
***λ*_1_ (×10^−3^mm^2^/sec)**	**W6** W29	**1.30±0.18** 1.36±0.17	**1.38±0.20** 1.39±0.22	***t*(10) = 3.272** *t*(10) = 1.439	**0.008** 0.181
***λ*_23_ (×10^−3^mm^2^/sec**)	W6 W29	0.73±0.11 0.78±0.11	0.74±0.14 0.75±0.17	T = 39; z = -0.533† T = 49; z = 1.423†	0.594**^†^** 0.155**^†^**

*Note.* ROIs correspond to the modelled damaged tract on the ipsilesional side and the respective contralateral tract. Values denote mean ± standard deviation (SD). For comparisons with normally distributed data, test statistics are represented using *t*(degrees of freedom), where *t* is the test statistic. For comparisons with non-parametric data, T is the test statistic and z is the standardized test statistic, or z-score. FA, fractional anisotropy; MD, mean diffusivity; *λ*_1_, primary eigenvalue, corresponding to AD; *λ*_23_, transverse eigenvalue, corresponding to RD; W6, week 6 post-stroke; W29, week 29 post-stroke. Bolded values indicate significance at *p* < .05.

**†**Wilcoxon signed-rank test.

### 3.2 Longitudinal change in diffusion indices along the ipsilesional tract

In the non-lesioned hemisphere, no significant differences were demonstrated between weeks 6 and 29 post-stroke for the FA, MD, *λ*_1_, and *λ*_23_, suggesting stability of the diffusion quantities across scans (see Inline Supplementary Table 1 for *p* values). This enabled us to use the ratios of diffusion metrics between the ipsilesional and contralesional side to study the longitudinal change along the modelled degenerating tract. The ratios for all parameters are presented in Table 3. The rFA along the ipsilesional tract decreased from the first to the second time point, albeit non-significantly. In comparison, the rMD, r*λ*_1_, and r*λ*_23_ all significantly increased over time.

**Table 3:** Diffusion parameters along the ipsilesional degenerating tract at two time points post-stroke in patients (n=11).

**Tract ROI Analysis**				
**Diffusion parameter**	**Time post-stroke**		**Test statistics**	**Group comparison, *P* value**
	**W6**	**W29**		**W6 vs W29**
**rFA**	0.94±0.13	0.91±0.13	*t*(10) = 1.602	0.140
**rMD**	**0.98±0.08**	**1.02±0.09**	***t*(10) = 2.942**	**0.015**
**r*λ*_1_**	**0.95±0.05**	**0.98±0.05**	***t*(10) = -2.479**	**0.033**
**r*λ*_23_**	**1.01±0.12**	**1.07±0.13**	***t*(10) = -3.206**	**0.009**

*Note.* Ratios between the ipsilesional and contralesional side in the ROI of the modelled damaged tract at two time points post-stroke. Values denote mean ± standard deviation. Test statistics are represented using *t*(degrees of freedom), where *t* is the test statistic. FA, fractional anisotropy; MD, mean diffusivity; *λ*_1_, primary eigenvalue, corresponding to AD; *λ*_23_, transverse eigenvalue, corresponding to RD; W6, week 6 post-stroke; W29, week 29 post-stroke. Bolded values indicate significance at *p* < .05.

### 3.3 Changes in diffusion indices along the ipsilesional tract at different distances from the primary infarction

Finally, to investigate the spatio-temporal changes in diffusion quantities along the modelled ipsilesional tract, we conducted a distance analysis on the rFA, rMD, r*λ*_1_, and r*λ*_23_ in five ROIs, comprising segments at 0–10, 10–20, 20–30, 30–40, and 40–50 mm from the primary lesion.

The repeated measures ANOVA showed a significant main effect of time for the rFA (*F*(1,10) = 8.31, *p* = .016), rMD (*F*(1,10) = 16.10, *p* = .002), r*λ*_1_ (*F*(1,10) = 9.88, *p* = .01), and r*λ*_23_ (*F*(1,10) = 25.00, *p* = .001). The rFA at week 29 post-stroke was significantly lower compared to week 6 across the five distances from the primary infarction. In contrast, the rMD, r*λ*_1_, and r*λ*_23_ were all significantly higher at week 29.

**Fig. 5:**
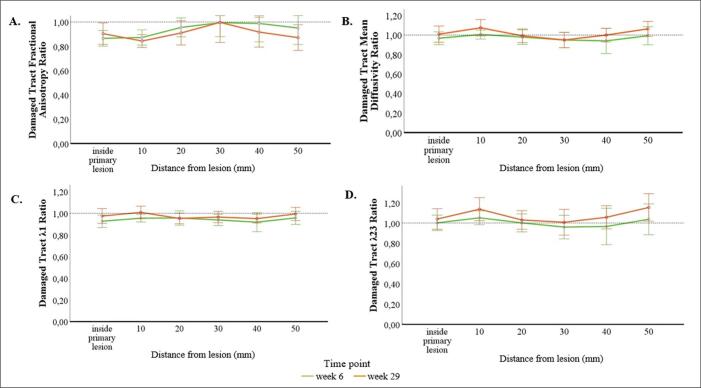
Ratios of the fractional anisotropy (A), mean diffusivity (B), primary eigenvalue (r*λ*_1_), corresponding to AD (C), and transverse eigenvalue (r*λ*_23_), corresponding to RD (D) between the ipsilesional and contralesional side in the ROIs of the primary lesion and at five distances along the modelled damaged tract at weeks 6 and 29 post-stroke in patients (n=11). Error bars denote 95 % confidence intervals (CIs).

Regarding the effect of distance, the rFA tended to be lower at week 29 post-stroke at the most proximal and distal segments from the primary lesion, whereas the rMD and ratios of eigenvalues exhibited the opposite pattern (see Inline Supplementary Table 2 for values at each distance). However, no main effect of distance or interaction between time and distance were found (Fig. 5).

## 4 Discussion

In the present study, we introduced a custom-developed method for modelling damaged fibers after ischemic stroke based on the lesion volumetric characteristics of individual patients. Modelling was done by superimposing segmented lesion volumes onto the tractograms of healthy subjects from a large tractography database. By adopting this approach, we could generate a prediction of the white matter regions where degeneration is expected to occur, addressing some of the caveats of standard procedures for ROI definition. Using the model prediction, we examined diffusion changes along the simulated streamlines to further elucidate the spatio-temporal progression of secondary degeneration.

### 4.1 Dynamic changes in the diffusion indices along the modelled white matter fibers damaged after stroke

To assess differences between the ipsilesional and contralesional hemisphere, we compared diffusion quantities along the fibers predicted to be damaged by the model and matched contralateral regions. At week 6 post-stroke, we found significantly decreased *λ*_1_ along the ipsilesional tract and the following non-significant tendencies for the remaining parameters: lower FA and diminished MD and *λ*_23_. At week 29, we documented significantly lower FA with a trend towards reduced *λ*_1_, preserved MD, and higher *λ*_23_ on the lesioned side. Although most comparisons did not reach statistical significance, diffusion indices changed in the anticipated directions. Given that secondary degeneration is characterized by a series of simultaneous events, we discuss significant and non-significant results concurrently to facilitate understanding of the molecular mechanisms underlying the observed changes.

The decrease in the *λ*_1_ at week 6 could be accounted for by the fragmentation of axons, which restricts the longitudinal displacement of water molecules (Kerschensteiner et al., 2005, Sun et al., 2008). After that, myelin sheaths start to disintegrate, causing an elevation in *λ*_23_. Subsequently, the axonal fragments are cleared by infiltrating microglia and diffusion in the parallel direction is re-established (Johnson et al., 1950, Lampert and Cressman, 1966), which may explain the increase in *λ*_1_ along the ipsilesional tract between week 6 and week 29 post-stroke. At the same time, the removal of the myelin debris enables water to diffuse more freely in the transverse direction (Song et al., 2003), causing *λ*_23_ to increase, which was observed between the two time points. These findings are in agreement with an earlier longitudinal investigation that demonstrated reduced *λ*_1_ at week 2, which elevated from 1 month to 3 months post-stroke, along with ongoing increases in *λ*_23_ (Yu et al., 2009). They suggest that axial diffusivity is a marker of axonal disruption, which precedes demyelination, whereas radial diffusivity changes reflect myelin loss, in line with evidence from animal studies (Beaulieu et al., 1996, Onuki et al., 2001, Song et al., 2005).

We also discovered lower FA along the ipsilesional tract at both time points with reduced MD at week 6 post-stroke that increased to the values of the contralateral tract by week 29. This fluctuation pattern is consistent with existing knowledge about the cellular mechanisms of secondary degeneration. More specifically, approximately 1 week following injury, MD decreases due to cytotoxic edema. Subsequently, cell lysis and degradation of tissue architecture enable water to diffuse freely again, causing an elevation in MD (Ahlhelm et al., 2002, Eastwood et al., 2003), which was demonstrated from week 6 to week 29 post-stroke. This is accompanied by a replacement of anisotropic microstructures with disorganized glial proliferation (Graham and Luntos, 2002), which may reduce FA. Despite the ongoing axonal and myelin disintegration, in the chronic stage of stroke, cellular debris and phagocytic microglia reintroduce barriers to water movement (Beaulieu et al., 1996). This may explain the preservation of MD along the ipsilesional tract relative to the contralesional tract at week 29. Comparable trends of diminished FA, with slightly raised MD from 1 month to 3 months (Yu et al., 2009) or unchanged MD 2 to 6 months following infarction (Werring et al., 2000), have formerly been reported.

Retrograde degeneration is also caused by axonal destruction and demyelination, but it starts at the infarct site and advances upstream (Jindahra et al., 2012). Previously, Liang and colleagues (2007) followed 12 patients with internal capsule lesions from week 1 to week 12 post-stroke. They interpreted the time-dependent degeneration in the fibers above the internal capsule as retrograde and the one along the thalamic radiation as anterograde. Similarly, we regard our findings as reflecting both types of secondary degeneration. To illustrate, for patients with pontine infarcts, the model prediction included the proximal portion of the pyramidal tract (including the centrum semiovale, internal capsule, and cerebral peduncle) where degeneration is deemed retrograde, and the medulla – where degeneration is considered anterograde (Liang et al., 2008).

Altogether, the exhibited differences between the ipsilesional and contralesional tracts were in the anticipated directions but many comparisons did not reach statistical significance. One possibility is that the approach for creating regions of interest may have included multiple tracts with different diffusion indices, adding substantial noise to our metrics. Although this could be the case in theory, we consider such a possibility unlikely due to several reasons. While baseline parameters of tracts do vary, the resulting effects would not affect longitudinal measurements in a within-subjects design considerably because diffusion changes related to secondary degeneration involve similar molecular mechanisms throughout the brain. In addition, the CST was largely represented in the prediction for most subjects, further limiting the contribution of inter-tract variations. A second putative explanation for the non-significant results could be the relatively mild-to-moderate lesion damage in our population. In this regard, it should be noted that the studied patients had different lesion locations with more prominent involvement of grey matter in some and white matter – in others. This can be relevant to our results since the pathological effects of grey matter injury could be exacerbated because damage to the cell soma causes the entire neuron to degenerate along with its axon. Nevertheless, the damaged streamlines model represented a group-mean, so the more pronounced effects from more severely affected individual cases could have been averaged out. Furthermore, our approach takes information about lesion extent into account in principle by modelling fewer damaged fibers for smaller lesions. However, some imprecision in the generated output for the smaller infarcts could have diluted the established effects. Another possibility is that the ipsilesional ROIs we analyzed comprised areas of crossing, where the preserved orientational coherence of the intact streamlines has offset the loss of anisotropy and increase in diffusivity (Pierpaoli et al., 2001). The latter underscores that the fibers’ structural morphology and spatial orientation should be taken into account when investigating secondary degeneration.

Furthermore, to assess the evolution of the longitudinal changes along the degenerating tract, we compared the ratios of diffusion indices between the ipsilesional and contralesional side. Along the damaged tract, the rFA decreased, whereas the rMD, r*λ*_1_, and r*λ*_23_ significantly increased over time. In keeping with previously inferred mechanisms of secondary degeneration, this is likely due to the further replacement of anisotropic microstructures distally from the lesion and the enlargement of the extracellular space (Beaulieu, 2002, Sen and Basser, 2005). More concretely, the clearance of the fragmented damaged axons by microglia enables water molecules to start diffusing in the longitudinal direction once again, causing diffusion parallel to fibers to rise. At the same time, the further degradation of the myelin sheaths and corresponding clearance of myelin debris without apparent axonal regeneration and new myelin formation increases the magnitude of diffusion in the perpendicular direction. These processes are indicative of axonal and myelin loss, as well as reduction in the orientational coherence of axons and axonal packing density (Song et al., 2003), and could explain the higher r*λ*_1_ and the ongoing elevation in r*λ*_23_. In turn, the changes in diffusion tensor eigenvalues might result in decreased rFA and increased rMD over time, reflecting the overall higher directionally independent diffusion, as observed from 1 month to 3 months post-stroke by Yu et al. (2009). Collectively, the demonstrated reduction in FA, accompanied by initial decreases in the parallel and subsequent increases in the transverse diffusivity, with small changes in MD, corroborate conclusions that secondary degeneration is characterized by fiber loss, as well as gliosis and extracellular matrix expansion (Pierpaoli et al., 2001, Thomalla et al., 2005, Werring et al., 2000).

### 4.2 Secondary degeneration along the damaged streamlines at different distances from the primary infarction

The used model also enabled an assessment of the changes in diffusion parameters along the ipsilesional tract at several distances from the primary lesion. Despite consensus that fiber degeneration affects adjacent regions of the axon sequentially, inconsistencies concerning its progressive nature and directionality prevail (Beirowski et al., 2005). To address this question, we defined five segments along the modelled streamlines at successive distances from the primary infarction. This approach complemented the tract ROI analysis and provided additional insight into the spatio-temporal development of secondary degeneration. While a longitudinal effect was revealed with lower rFA and significantly higher rMD, r*λ*_1_, and r*λ*_23_ at week 29 post-stroke, the established differences did not vary significantly with distance from the primary lesion. The progressive decrease in anisotropy and increase in diffusivity across the different segments of the damaged tract support the notion that degeneration affects the entire fiber’s length (Liang et al., 2008). Still, some non-significant variation was observed at different locations along the ipsilesional tract with lower rFA and elevated rMD, r*λ*_1_, and r*λ*_23_ most proximally and remotely from the primary infarction. This fluctuation pattern may indicate more pronounced diffusion changes in these areas as existing literature has suggested that axonal degeneration is region-specific (Burzynska et al., 2010) and contingent upon factors, such as fiber type (Martinez and Canavarro, 2000), axonal diameter and packing density (Takahashi et al., 2002), membrane permeability (Beaulieu, 2002), and axonal arrangement within a voxel (Jones et al., 2013). Another potential explanation concerns inherent variations in white matter anatomy, particularly in areas of crossing fibers where tensor-derived scalar measures could reflect the degree of fiber dispersion (Dell’Acqua and Tournier, 2019), rather than constitute a reliable anatomical correlate of a clinical condition. Given that direct histopathological evidence cannot be provided, future research should further clarify whether areas most proximal and distal to a lesion are more susceptible to the influence of secondary degenerative processes.

### 4.3 Methodological considerations

Several limitations warrant consideration. It should be outlined that the tensor model provides a simplified description of the diffusion process compared to the underlying histology (Alexander et al., 2002, Tuch et al., 2002). The ROIs we analyzed largely overlapped with the CST, which has highly oriented fibers suitable for DTI assessment (Yu et al., 2009). Nevertheless, most white matter tracts do not remain within single-fiber voxels over their entire path (Jeurissen et al., 2013). Hence, it is likely that the model prediction comprised regions with contributions of crossing fibers, such as the rostral pons (Pierpaoli et al., 2001) or anterior limb of internal capsule (Schmahmann and Pandya, 2006) where secondary degeneration effects may be underestimated. Using a higher-order tractography technique in the future will provide a more sensitive measure of the stroke-induced changes at the intravoxel level (Dell’Acqua and Tournier, 2019). Second, control subjects in the database were not age-matched to the patients, which in theory might have added noise to the prediction of the streamlines to be damaged by the lesions. Although we attempted to minimize such effects by adjusting the normalization procedure to account for age-related effects (i.e., ventricular size), we cannot exclude some residual bias. Ideally, separate databases should be created that fully match the demographic details of the experimental population. Third, patients were allocated to the favorable prognosis group within the recruitment trial where ability to carry out voluntary flexion–extension and grasping movements was required, which could have introduced selection bias. Another consideration is the lack of baseline data and limited sample size, which make it difficult to assess any diffusion alterations prior to week 6 post-stroke. These shortcomings may compromise the generalizability of our results, particularly to patients with more severe strokes. Lastly, it is worth noting that an examination of tract-related metrics in relation to neuropsychological measures of upper limb motor recovery was beyond the scope of this study. Earlier investigations have discovered associations between FA values and clinical assessments, such as the NIHSS and Motricity Indices (Thomalla et al., 2004, Yu et al., 2009), and Fugl–Meyer (FM) scale (Liang et al., 2007). However, they linked their motor function measurements to diffusion parameters at earlier time points (within 2 days to 12 weeks post-stroke) when most spontaneous neurobiological recovery is thought to occur (Kwakkel et al., 2006). Future studies using longer observation periods, more intermediate time points for comparison, larger populations with varying degrees of motor impairment, combined with resting-state or task-related brain activation analyses, would help further disentangle the spatio-temporal development of secondary degeneration and its relationship to functional recovery.

### 4.4 Implications and conclusion

Notwithstanding these concerns, the current study has important implications. First, a prominent contribution is the introduction of a non-invasive approach for modelling damaged fibers based on the lesion volumetric characteristics of individual patients. Despite the lesion heterogeneity found within our sample, results revealed a strong link between the simulated streamlines and the motor deficit that patients presented with at stroke onset. This demonstrates that our method can effectively handle the inter-subject variability in infarct size and location, common to stroke.

Second, the established relationship between the modelled damaged fibers and the upper limb paresis observed in patients implies that white matter degeneration can be used as a reliable predictor of stroke-related functional outcomes (Lin et al., 2019), in agreement with existing research. To illustrate, prior investigations among stroke patients have reported that the extent of CST damage and not infarct size *per se* are crucial determinants of post-stroke motor ability (Sterr et al., 2010, Zhu et al., 2010). Similarly, Chen et al. (2000) examined the impact of brain lesion factors on motor measures in hemiplegic stroke patients and documented that patients with poorer brain lesion profiles had worse motor recovery regardless of lesion location, possibly due to the involvement and destruction of the CST due to Wallerian degeneration. Building on such evidence about the key prognostic value of white matter degeneration, our method can provide a comprehensive set of affected voxels along modelled fibers for finding correlations with behavioral symptoms. In turn, this can assist early prediction of stroke-related impairment with the potential to facilitate hospital discharge planning (Kwakkel et al., 2011) and help stratify patients, allowing to optimize patient-specific prognosis (Selles et al., 2021) and rehabilitation goals (Kwakkel et al., 2015). An additional merit of our modelling procedure is that it can enhance associations between lesions and white matter disruptions with respect to more complex functions, such as cognitive ability. We are therefore confident that our approach has promising future applications, both in neurological research and clinical practice.

To conclude, we used a fiber modelling method to quantify white matter damage after ischemic stroke and explored the dynamic development of secondary degeneration along the simulated streamlines. Our findings suggest that secondary degeneration spreads along the entire length of a damaged tract. Adopting more sophisticated diffusion imaging techniques in future examinations can further map the complex configurations of the living and diseased human brain.

## Ethical approval

The study protocol and all procedures involving human participants were approved by the local ethics committee (Medical Ethics Review Committee of Leiden University Medical Center (No. P08.035) and Dutch Central Committee on Research Involving Human Subjects (CCMO: No. NL21396.058.08)). The EXPLICIT-stroke trial was registered in the Dutch Trial Registry (NTR, www.trialregister.nl, TC1424).

## CRediT authorship contribution statement

**Ivana Kancheva:** Conceptualization, Data curation, Formal analysis, Investigation, Methodology, Validation, Visualization, Writing – original draft. **Floor Buma:** Conceptualization, Data curation, Methodology, Writing – review & editing. **Gert Kwakkel:** Conceptualization, Data curation, Methodology, Resources, Writing – review & editing, Project administration, Funding acquisition. **Angelina Kancheva:** Methodology, Writing – review & editing. **Nick Ramsey:** Conceptualization, Resources, Project administration, Funding acquisition. **Mathijs Raemaekers:** Conceptualization, Data curation, Formal analysis, Investigation, Methodology, Resources, Software, Visualization, Writing – review & editing, Supervision, Project administration.
