# Supplementary material for: Investigating secondary white matter degeneration following ischemic stroke by modelling affected fiber tracts
Source: Neuroimage Clin. 2022 Jan 22;33:102945. doi: 10.1016/j.nicl.2022.102945 (PMC8829801; doi:10.1016/j.nicl.2022.102945)
Supplement: Supplementary data 1 [file mmc1.docx]

**Investigating Secondary White Matter Degeneration Following Ischemic Stroke by Modelling Affected Fiber Tracts**

**Appendix A. Supplementary Data**

**Inline Supplementary Material:**

| **Supplementary Table 1.** Comparisons of diffusion parameters on the ipsilesional and contralesional side between two time points post-stroke in patients (n=11). | | | | |
| --- | --- | --- | --- | --- |
|  | **Time post-stroke** | | **Test  statistics** | **Group comparison,  *P* value** |
| ***Ipsilesional side*** |  |  |  |  |
| **Diffusion parameter** | **W6** | **W29** |  | **W6 vs. W29** |
| **FA (dimensionless)** | 0.40±0.07 | 0.39±0.06 | *t*(10) = 2.198 | 0.053 |
| **MD (×10^−3^mm^2^/sec)** | 0.92±0.12 | 0.97±0.12 | *t*(10) = -2.208 | 0.052 |
| ***λ*_1_ (×10^−3^mm^2^/sec)** | 1.30±0.18 | 1.36±0.17 | *t*(10) = -1.997 | 0.074 |
| ***λ*_23_ (×10^−3^mm^2^/sec)** | **0.73±0.11** | **0.78±0.11** | **T = 60; z = -2.401^†^** | **0.016^†^** |
| ***Contralesional side*** |  |  |  |  |
| **FA (dimensionless)** | 0.43±0.06 | 0.43±0.07 | T = 39; z = -.533**^†^** | 0.594**^†^** |
| **MD (×10^−3^mm^2^/sec)** | 0.95±0.15 | 0.96±0.18 | T = 34; z = -.089**^†^** | 0.929**^†^** |
| ***λ*_1_ (×10^−3^mm^2^/sec)** | 1.38±0.20 | 1.39±0.22 | *t*(10) = -.387 | 0.707 |
| ***λ*_23_ (×10^−3^mm^2^/sec)** | 0.74±0.14 | 0.75±0.17 | T = 32; z = -.089**^†^** | 0.929**^†^** |
| *Note.* ROIs correspond to the modelled damaged tract on the ipsilesional side and the respective contralateral tract. Values represent mean ± standard deviation (SD). For comparisons with normally distributed data, test statistics are represented using *t*(degrees of freedom), where *t* is the test statistic. For comparisons with non-parametric data, T is the test statistic and z is the standardized test statistic, or z-score. FA, [fractional anisotropy](https://www.sciencedirect.com/topics/medicine-and-dentistry/fractional-anisotropy); MD, mean diffusivity; *λ*_1_, primary eigenvalue, corresponding to AD; *λ*_23_, transverse eigenvalue, corresponding to RD; W6, week 6 post-stroke; W29, week 29 post-stroke. Bolded values indicate significance at *p* < .05.  **†**Wilcoxon signed-rank test | | | | |

| **Supplementary Table 2.** Diffusion parameters in six regions of interest at two time points post-stroke in patients (n=11). | | | | | | | |
| --- | --- | --- | --- | --- | --- | --- | --- |
| **Distance-based ROI analysis** | | | | | | | |
| **Time   post-stroke** | | **Primary   lesion** | **1** | **2** | **3** | **4** | **5** |
| ***rFA*** | | | | | | | |
| **W6** | 0.87±0.10 | | 0.87±0.09 | 0.96±0.12 | 0.99±0.17 | 0.99±0.23 | 0.95±0.20 |
| **W29** | 0.90±0.13 | | 0.84±0.08 | 0.91±0.15 | 1.00±0.25 | 0.92±0.18 | 0.87±0.16 |
| ***rMD*** | | | | | | | |
| **W6** | 0.97±0.10 | | 1.00±0.07 | 0.98±0.11 | 0.95±0.12 | 0.94±0.19 | 0.99±0.14 |
| **W29** | 1.01±0.12 | | 1.07±0.13 | 0.99±0.10 | 0.95±0.12 | 1.00±0.10 | 1.06±0.12 |
| ***rλ_1_*** | | | | | | | |
| **W6** | 0.93±0.09 | | 0.95±0.05 | 0.96±0.10 | 0.94±0.08 | 0.92±0.13 | 0.96±0.09 |
| **W29** | 0.97±0.10 | | 1.01±0.09 | 0.95±0.07 | 0.97±0.08 | 0.95±0.06 | 0.99±0.09 |
| ***rλ****_23_* | | | | | | | |
| **W6** | 1.00±0.11 | | 1.05±0.10 | 1.00±0.13 | 0.96±0.17 | 0.97±0.27 | 1.04±0.23 |
| **W29** | 1.04±0.15 | | 1.14±0.17 | 1.03±0.14 | 1.01±0.19 | 1.06±0.17 | 1.15±0.20 |
| *Note.* Ratios of the fractional anisotropy (rFA), mean diffusivity (rMD), primary eigenvalue (r*λ*_1_), corresponding to AD, and transverse eigenvalue (r*λ*_23_), corresponding to RD, between the ipsilesional and contralesional side in the ROIs of the primary lesion and at five segments along the modelled damaged tract. Arabic numbers (1, 2, 3, 4, 5) denote distances from the primary infarction, i.e., 0-10, 10-20, 20-30, 30-40, 40-50 mm, respectively. Values represent mean ± standard deviation (SD). W6, week 6 post-stroke; W29, week 29 post-stroke. | | | | | | | |
